# Several insights into the preprocessing of electrograms in atrial fibrillation for dominant frequency analysis

**DOI:** 10.1186/s12938-016-0157-2

**Published:** 2016-04-12

**Authors:** Wenhai Li, Cuiwei Yang, Yanlei Wang, Dexi Wang, Ying Chen, Zhong Wu

**Affiliations:** Department of Electronic Engineering, Fudan University, Shanghai, 200433 China; Key Laboratory of Medical Imaging Computing and Computer Assisted Intervention of Shanghai, Shanghai, 200433 China; Shanghai Engineering Research Center of Assistive Devices, Shanghai, 200433 China; Department of Electronic Science and Engineering, Nanjing University, Nanjing, 210046 Jiangsu China; Department of Cardiovascular Surgery, West China Hospital, Sichuan University, Chengdu, Sichuan 610041 China

**Keywords:** Atrial fibrillation, Dominant frequency, Preprocessing, Down-sampling, Electrograms

## Abstract

**Background:**

Dominant frequency (DF) analysis of atrial electrograms has become an important method in characterizing atrial fibrillation (AF). As a classic method, Botteron’s approach is widely used in the preprocessing of frequency analysis during AF. It includes three steps: (1) band-pass filtering at 40–250 Hz, (2) absolute value, and (3) low-pass filtering at 20 Hz. This paper aims to expound the necessity and adjustability of each step.

**Methods and results:**

Unipolar epicardial mapping signals were recorded during AF from eight mongrel dogs with cholinergic AF model. Episodes of these data were randomly selected to evaluate the impact of different pass bands and the necessity of low-pass filtering with 20 Hz cutoff frequency. Each episode of AF signal is 5 s long with a sampling rate of 2 kHz. Simulated electrograms were adopted to discuss the role of taking absolute value. Furthermore, direct spectral analysis method (FFT et al.) is compared with Botteron’s preprocessing approach. According to our statistical analysis, the pass band of 40–250 Hz was not the best, while 20–100 Hz presented the high accuracy rate of DF. From the comparing result of direct FFT without Botteron’s approach we deduced that the rectification of absolute value was meaningful for the fundamental atrial frequency. The final step, 20 Hz low-pass filter can completely be omitted in DF analysis. In consideration of the demand for real-time distribution of DF in clinical or experimental situations, down-sampling method and the impact of ventricular artifacts on DF was also discussed.

**Conclusion:**

In the actual application of the three preprocessing steps, the pass band selection of band-pass filter can be adjusted and the rectification of taking absolute value is important. Nevertheless, the final step of 20 Hz low-pass filter is totally unnecessary. In real-time signal processing situations, taking down-sampling method and ignoring the ventricular artifacts can also have high performance in DF analysis of atrial electrograms.

## Background

Atrial fibrillation (AF) is currently a common clinical arrhythmia with a high morbidity and mortality but the electrophysiological and pathological mechanisms of AF are still not well understood [1–5]. The analysis of the atrial electrical activity plays an important role to reveal the underlying electrophysiological mechanisms responsible for AF’s initiation, maintenance and perpetuation. In recent studies, sites with high dominant frequency (DF) are regarded as drivers of AF [6–8] and therefore become the targets for AF ablation [7, 9, 10]. Hence, in ECG mapping system dominant atrial frequency is a key parameter for the analysis of AF [5, 11, 12].

For atrial activation, DF is the frequency component which marks the atrail dominant rhythm. In the frequency domain it is the maximum fundamental peak [8, 9, 13]. For example, if the myocardial tissue excited 30 times in 10 s, the DF should be 3 Hz, and the amplitude of 3 Hz should be the largest peak in the frequency domain. Botteron’s approach is the most widely employed preprocessing approach to estimate the dominant atrial rate. The preprocessing steps are: (1) band-pass filtering at 40–250 Hz, (2) absolute value, and (3) low-pass filtering at 20 Hz [8, 9, 14–16]. After these three steps, DF is the maximum peak’s corresponding frequency in the power spectrum wave. According to ISI Web of Knowledge, the number of research with title containing “dominant frequency” is about 140, and up to date the works of Botteron [14, 15] have been cited 225 times [17, 18]. When Botteron first proposed this method in 1995 and 1996 [14, 15], his approach was not for seeking the DF, but for the correlation calculation. However, it is now regarded as a classic preprocessing method [8, 9, 13, 16, 17].

In 2014 Castells et al. explained the theoretical basis of the preprocessing process [17], but they didn’t introduce what should be noted on real data analysis. Furthermore, in recent years, some researchers do DF analysis not in accordance with the above three steps [12, 19, 20]. In this paper the role and the necessity of each step in Botteron’s approach are addressed by using real data recorded from the 128-channel unipolar epicardial mapping system. For real time DF analysis, we also put forward two proposals to save the calculation time: reducing the sampling frequency and ignoring the ventricular artifacts.

## Methods

In this study, we devised animal experiments to collect real-world data using living canine subjects with cholinergic AF model. The canine heart was exposed after thoracotomy and suspension of the pericardium. 8 flexible patches containing a total of 128 unipolar electrodes were carefully attached to right atrium, left atrium and the root of pulmonary veins. AF was induced by rapid pacing (with a frequency of 20 Hz) at the atrial appendages of the canine heart with an intravenous injection of acetylcholine (see details of experimental procedures and mapping sites in [21] and [22]). Then real data were recorded at a sampling frequency of 2 kHz from the 128-channel unipolar epicardial mapping system developed by the Electrophysiology Laboratory in Fudan University [21, 22]. The hardware of our mapping system included amplifying and filtering, and the hardware filters were a notch filter at 50 Hz and a band pass filter (BPF) at 3–500 Hz. The software which contain signal processing and analyzing were implemented with MATLAB (Mathworks Inc.). 8 dogs (weight 13.4 ± 2.9 kg) was used, all these data formed a signal database in the Electrophysiology Laboratory of Fudan University, and all the AF data used in this study were randomly selected from this database.

### Dominant frequency and Botteron’s approach

**Fig. 1:**
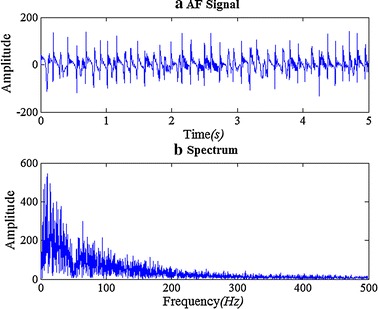
The unipolar epicardial AF signal and its spectrum. **a** A segment of epicardial AF signal collected from a cholinergic AF model dog. **b** The spectrum of the AF signal, note that a 50 Hz notch filter is used when collected the signal

Figure 1 shows a unipolar epicardial AF signal and its spectrum. DF is an important frequency domain index, which often detected as the frequency with the maximum power in the power spectrum. Preprocessing may be needed for a more accurate DF result. Botteron’s approach is just the classic preprocessing method, its preprocessing steps are: (1) band-pass filtering at 40–250 Hz, (2) absolute value, and (3) low-pass filtering at 20 Hz.

**Fig. 2:**
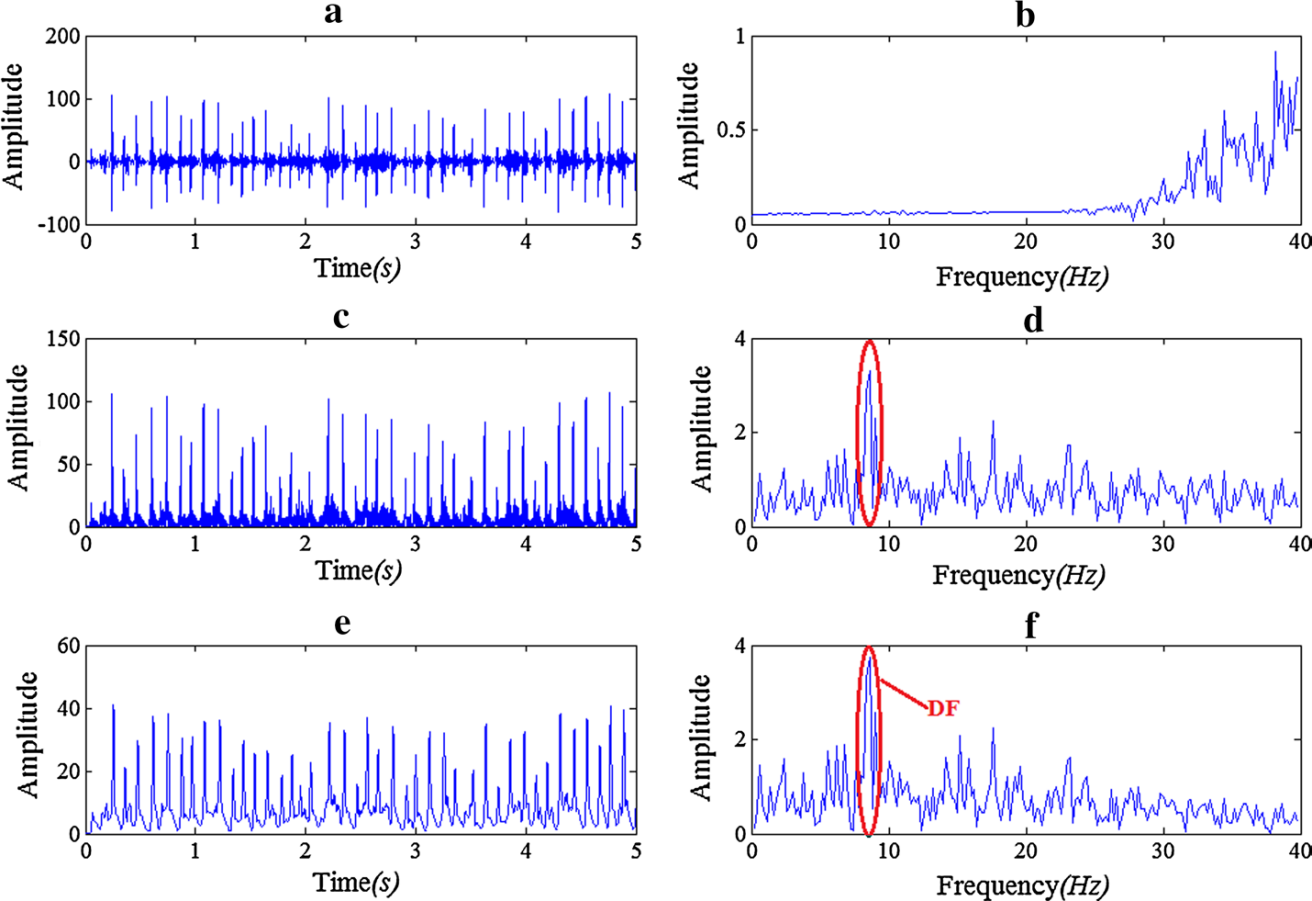
The signal wave and its spectrum after the preprocessing steps of Botteron’s approach. **a**, **b** Band-pass filtering at 40–250 Hz; **c**, **d** absolute value, and **e**, **f** low-pass filtering at 20 Hz. The original signal is showing in Fig. 1

Figure 2 shows each step of Botteron’s approach and the signal’s spectrum after each step. The spectrum is based on fast Fourier transform (FFT). A Hamming window is used before FFT.

Each of the above three steps has its specific role. But considering the reason that this preprocessing algorithm was not for DF analysis, but for the correlation calculation when Botteron first proposed it in 1995, these three steps could be adjusted if used in seeking for DF, especially during AF.

### Adjust the parameters of band-pass filter

Band-pass filter (BPF) is often used to eliminate noise and interference, such as baseline drift (caused by breathing or beating of the heart) and high frequency noise [23]. Botteron first used this preprocessing step to calculate the cross-correlation of signals, especially for the activation pattern, since the components between two activation intervals may influence the results [14, 15] and the spectral content of those components are below 40 Hz [17]. Nevertheless, due to the fact that DF is within 20 Hz, such “interference” has smaller impact on DF than on cross-correlation and therefore the selection of pass band may vary with the actual situation.

In order to get an effective BPF, we firstly analyze the energy distribution of epicardial mapping signals during AF in frequency domain. As Fig. 1 shows, the energy of epicardial AF signal is concentrated in 10–100 Hz, which is different from some previous studies [24, 25]. To improve the performance, we could make an appropriate adjustment for the range of 40–250 Hz.

In this study, all the band pass filters are FIR filter and we adjusted the cutoff frequency of the filter and then compared the DF results. The statistical analysis of DF results was also done with different pass band.

### Taking absolute value

Taking absolute value in Botteron’s approach is also known as the rectification to restore the low frequency components after band-pass filtering [17]. During this process, the DF which reflects the main rhythm of signal will appear and be highlighted. After taking absolute value, the amplitude of the direct current (DC) component is the largest and the maximum peak will appear at 0 Hz. Then, DF is shown as the second maximum peak. For the theoretical explanation of the effect by taking absolute, you could also see the article [17], which is to find out the changes in the spectrum area by the analysis of phase change. Here we will illustrate the role of absolute value from another perspective.

**Fig. 3:**
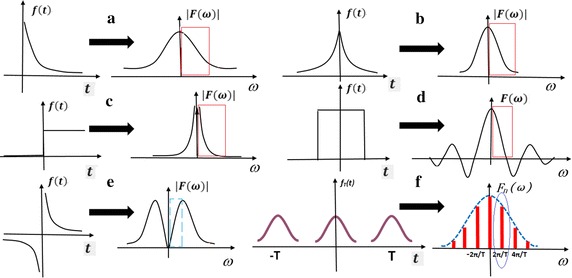
Spectrum of common waveforms

Due to myocardial cells’ fast depolarization, the epicardial ECG signal will have a steeper slope when the tissue is exciting. The shape of a single ECG after taking the absolute value is similar to that of waveform *f*(*t*) in graph (a) or (b) of Fig. 3, some may also be similar to the waveform shape in graph (f). From a theoretical point of view, one episode of epicardial ECG signal can also be obtained (mainly superposition) by the linear combination of waveforms in (a), (b), (c), (d) and (f). The spectrum of these five waveforms all decreases their amplitude when frequency increasing from 0 Hz as the red box in Fig. 3 shows. In case the waveform occurs periodically, the spectrum becomes discrete spectrum, as shown in Fig. 3f. The decreasing envelope shape, combined with discrete spectral line appears at every other *f*_0_ (*f*_0_ = 1/*T*). We can clearly see that, except 0 Hz, the first line has the maximum peak and is corresponding to the frequency of any periodical signal. This is the reason why the DF could reflect signal’s rhythm (period or frequency) and accords with the largest peak in the spectrum.

The role of taking absolute value is very important. When a signal has both positive part and negative part (before taking absolute value), as graph (e) shows in Fig. 3 [e.g. the ECG waveform in Fig. 6 is close to (c) plus (e) in Fig. 3], its spectrum would not show a single reduction trend from 0 Hz. Thus when the first spectral line falls into the decreasing area, it is still the largest peak, which can accurately correspond to the DF. Otherwise, when the first discrete line falls into the area before the maximum peak (the blue box in Fig. 3e), the maximum peak line may no longer corresponding to *f*_0_.

### Low-pass filtering at 20 Hz

Botteron proposed the preprocessing at first (1995) for the correlation algorithm [14, 15]. In fact, after 20 Hz low-pass filtering all kinds of excited waveforms become similar with each other [9]. This could get rid of different waveforms’ effect caused by different atrial positions [14]. The effect of this low-pass filter (LPF) is just filtering out the components beyond 20 Hz, so that the waveform in the time domain would be more similar to the sine wave shape. However, as for seeking DF, low-pass filtering at 20 Hz is actually not necessary because the rhythm of atrial signal is within 20 Hz.

In general, after the previous two steps: band-pass filtering and rectification, the DC component of the signal is increased, the maximum peak in the frequency domain is at 0 Hz and the frequency at the second largest peak is the DF, which is within 0–20 Hz.

### Without Botteron’s preprocessing steps

Some researchers do DF analysis not in accordance with the three steps of Botteron’s approach [12, 19, 20]. Their method is just directly doing spectral analysis based on FFT.

To illustrate and compare the DF result of the direct FFT method and Botteron’s approach, we marked the two methods as: method 1-directly do spectral analysis (FFT) and the DF result is denoted by DF_1_; method 2-first 20–100 Hz band-pass filtering, subsequently taking absolute value, finally do spectral analysis (FFT) and getting the DF result DF_2_. Then the relative ratio R was defined as:

$$R = \frac{{\left| {DF_{1} - DF_{2}} \right|}}{DF_{2}}$$

If R < 0.1, the results of these two methods were regarded identical. On the other hand if R > 1, we think the results were completely different from each other.

### Evaluating the effect of sampling frequency on DF

Down-sampling is known as the decimation in digital signal processing [26]. Before decimation, LPF is needed to mitigate aliasing distortion. *M*-times decimation to the original discrete-time signal (sampling frequency *f*_1_) is just taking one data after every *M*-*1* data and getting the sampling frequency of *f*_1_/*M* [27, 28]. After down-sampling the signal could lose high-frequency components and the maximum frequency *f*_*max*_ < *f*_1_/(2 *M*). Therefore, after down-sampling high-frequency components are removed but low-frequency components are kept.

Different acquisition system may have different sampling frequency. Low sampling frequency could benefit the real-time performance of DF mapping. Here we used the down-sampling method on our 128-channel epicardial signals to construct different sampling frequency and evaluated the influence of sampling frequency on DF.

### The influence of ventricular far-field depolarization on DF

The crosstalk from the far-field ventricular potential is also apparent when collecting the atrial signal and we can call it ventricular artifact. Removing the crosstalk from ventricle may often take away a portion of the atrial activation information and lead to a distorted signal. So, whether it is necessary to remove the ventricular artifact when doing the DF analysis during AF is worth considering.

Ng et al. has especially analyzed the impact of the ventricular far-field depolarizations (VFDs) and found VFDs significantly affected DFs [8]. However, they ignored the fact that the impact on dominant atrial frequency was limited when the ventricular amplitude was small. During endocardial and epicardial mapping, the electrodes are directly attached to the atrial tissue to collect signal and the amplitude of atrial signal is much larger than the amplitude of VFDs (only in the area near ventricle the amplitude will be larger).

In this study, we employed the ventricular reference signals from the apex of the heart, and introduced a least mean square adaptive filter with noise canceller model to remove the VFDs [29]. Then we did DF analysis and compared it with the DF which from the same signal but with VFDs.

## Results

### Adjusting the parameters of band-pass filter

**Fig. 4:**
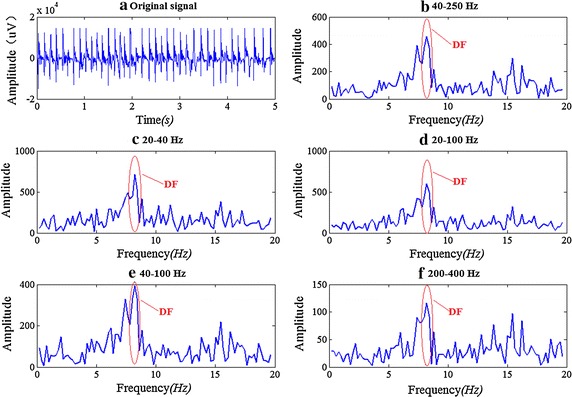
Effects of different pass band ranges on seeking DF. **a** The original signal; **b** the spectrum and DF of the signal after band-pass filtering of 40–250 Hz; **c** the spectrum and DF of the signal after band-pass filtering of 20–40 Hz; **d** the spectrum and DF of the signal after band-pass filtering of 20–100 Hz; **e** the spectrum and DF of the signal after band-pass filtering of 40–100 Hz; **f** the spectrum and DF of the signal after band-pass filtering of 200–400 Hz. Absolute value and LPF at 20 Hz are taken after band-pass filtering in* graph* **b**–**f** and the DF is marked out in these figures

Figure 4 shows the result of DF by band-pass filters with different pass band ranges, we can see that for the pass band of 20–40, 20–100, 40–100, 200–400 Hz, the DFs are the same with that of 40–250 Hz. What’s more, as Fig. 4b–f show that there is no big change in the spectral waveform of 0–20 Hz.

**Fig. 5:**
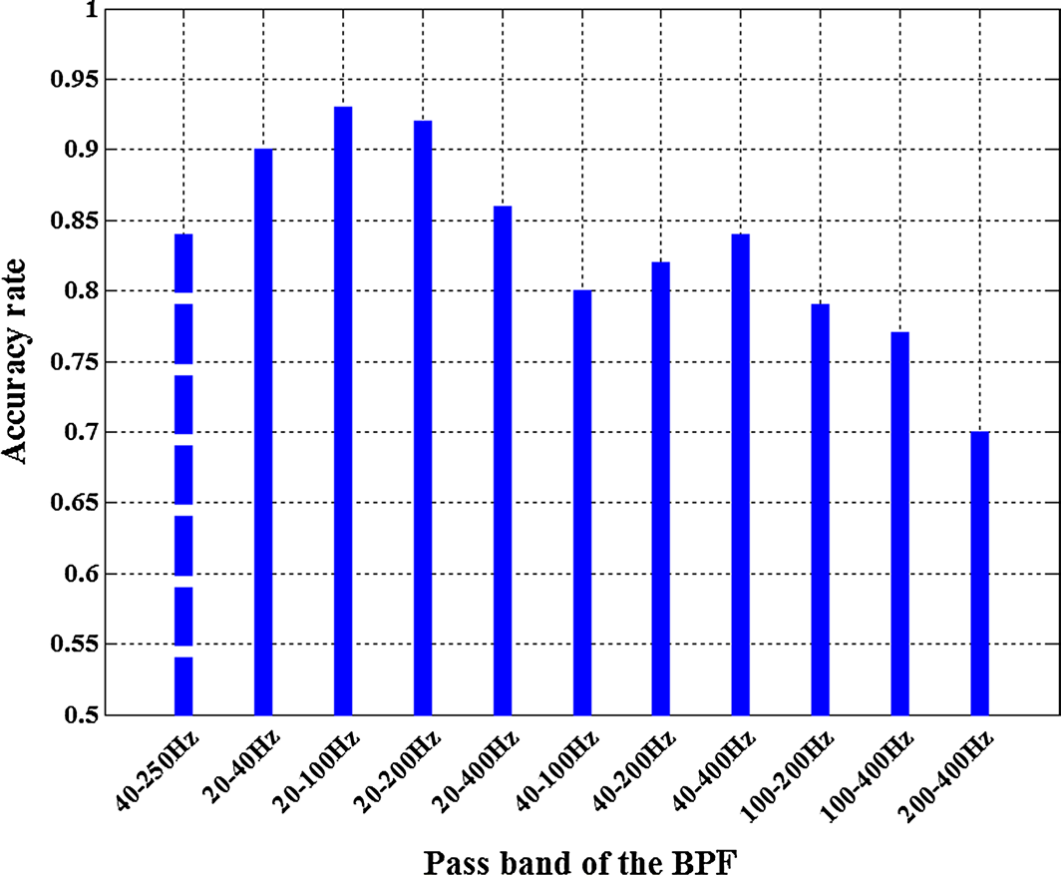
The DF accuracy rate by using different pass band BPF. The accuracy rate of classical 40–250 Hz is shown in the* left* with *dotted line* to getting a good comparison

However, the DF results may not be always correct when using the above different band pass ranges. To illustrate whether the 40–250 Hz BPF is the best, we did statistical analysis of DF results with different passband. We randomly selected 100 episodes of AF signal from our epicardial AF signal database (the total number of activation beat is about 3000). Each episode of AF signal is 5 s long (see [8]) with a sampling rate of 2 kHz. For each episode, we used the manual marking method (clinical experts marked the excitement, and then we divided number of marks by time interval) as the golden standard to evaluate whether the DF value was correct. Figure 5 shows the statistical result. The DF as determined from FFT is within 5 % error of the manual marking calculation. For example, if the DF determined from FFT is between 4.75 and 5.25 Hz, we think it is the same as the manual marking calculation of 5 Hz. It can be seen that 84 episodes of signal (84 %) get the same DF value with manual method when the pass band is 40–250 Hz. But when the pass band is selected as 20–100 Hz, the accuracy rate is 93 %. What’s more, 20–40 and 20–200 Hz are all better than 40–250 Hz. It could be concluded from the statistical result that 20–100 Hz is the best pass band when the energy of epicardial AF signal is concentrated in 10–100 Hz (see Fig. 1).

### Taking absolute value is important after BPF

**Fig. 6:**
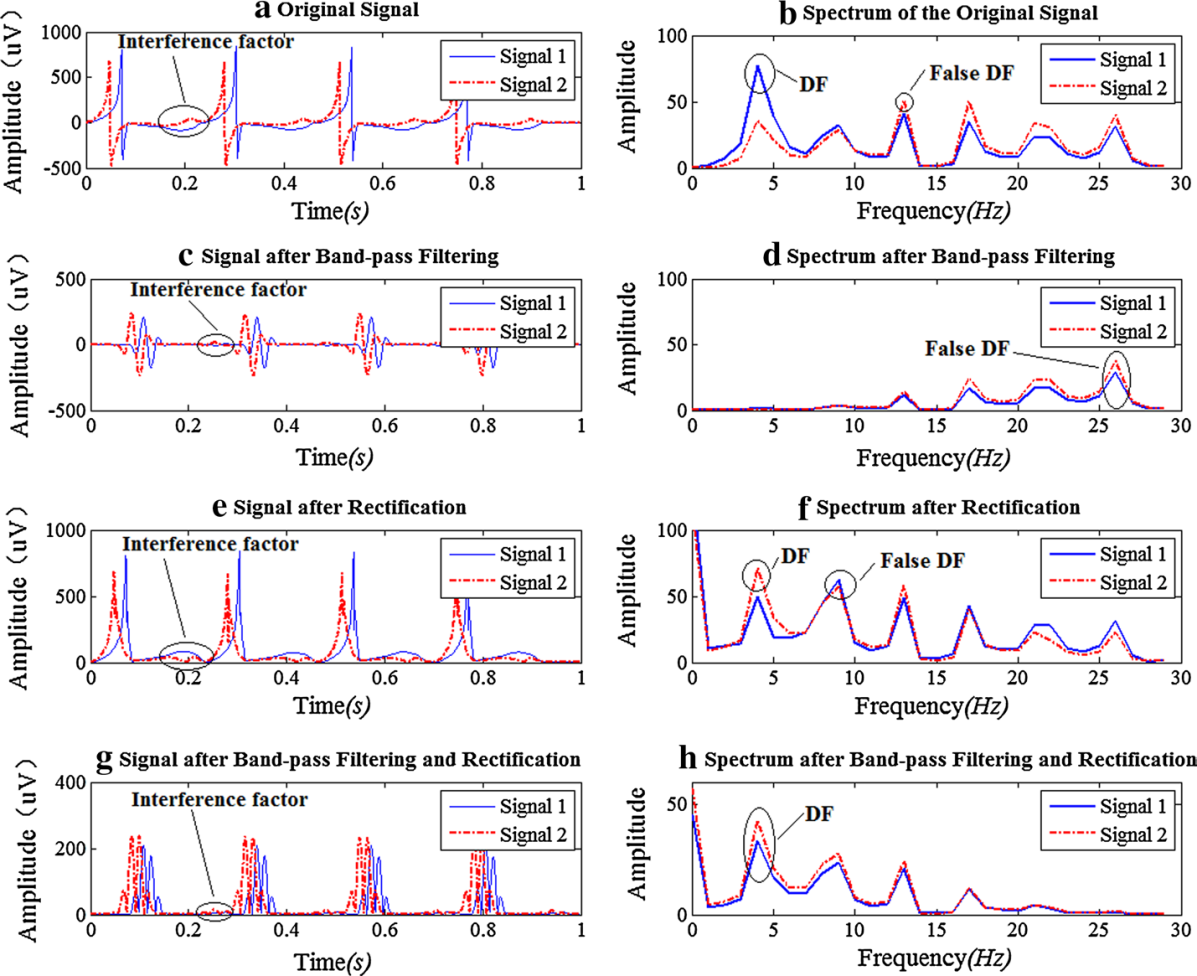
The comparison by taking different preprocessing steps in two simulated ECG signals. The *left column* shows the signals’ morphology in time domain, and the *right column* shows the corresponding spectrum and DF

Here we give an example to show the important step of taking absolute value. Turning to the pattern of the simulated ECG signal 1 and 2 in Fig. 6a. These two signals both have four activations within 1 s, so the DF should be 4 Hz. If we directly do FFT, signal 1 can get a correct DF. Conversely, signal 2 cannot achieved a correct one (as shown in Fig. 6b). In particular if we just do BPF (40–100 Hz), no signal will get a right value of DF (see Fig. 6d). As a result when we adopt the only step of taking absolute value, signal 2 can get a correct DF, while signal 1 cannot (as shown in Fig. 6f). It’s because signal 2 has a significant low-frequency interference component and with rectifying it is reduced. On the contrary, with rectifying, there appears a significant interference in signal 1 (the interference have been figure out in Fig. 6a, e by oval marks). As for BPF and then rectification, signal 1 and signal 2 both can get the true DF (see Fig. 6g, h).

### Unnecessary LPF of 20 Hz

**Fig. 7:**
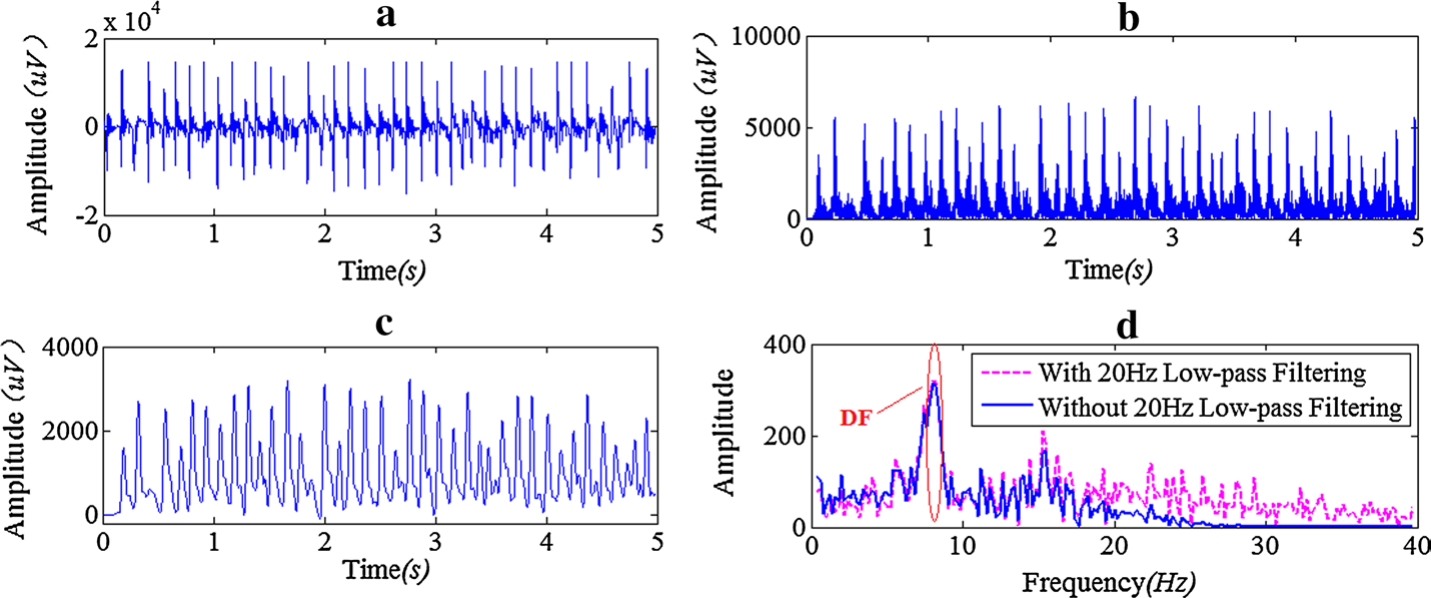
The contrast of the DF results with and without 20 Hz low-pass filtering. **a** Original signal, fs = 2 kHz; **b** signal after 40–250 Hz band-pass filtering and rectification; **c** signal after 20 Hz low-pass filtering; **d** frequency spectrum and DF. DC component has been moved out in* graph* **d**

Figure 7 shows the spectrum and DF result with and without low-pass filtering on the AF signals recorded by our epicardial mapping system with 5 s duration, from which we can see that a 20 Hz LPF actually has no effect on the evaluation of DF.

### Without Botteron’s preprocessing steps

**Fig. 8:**
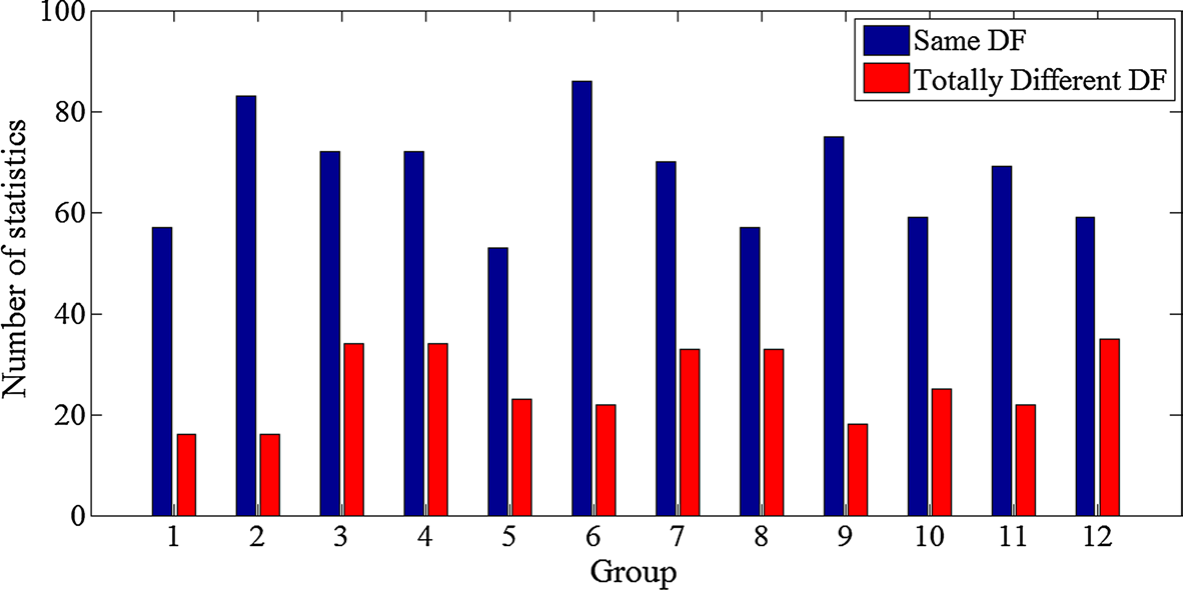
The comparison of the DF results by direct FFT method and Botteron’s classical method. Each group has 128 signals, count the number that the signal has same DF(R < 0.1) by the two method and the number that the signal has totally different DF(R > 1)

To illustrate and compare the DF result of the direct FFT method and FFT after Botteron’s preprocessing, we randomly selected 12 segments of atrial epicardial mapping data of AF. Each segment of data has 128 channels signals lasting 5 s with sampling frequency fs = 2 kHz, without any other preprocessing except hardware filter (see “Methods”). The result is shown in Fig. 8.

It can be seen that nearly half number of the channel’s signal obtained the same DF value by these two methods, which indicating that the direct FFT method could really get the correct DF value for some signals. It is worth noting that, all the signals are without any preprocessing. If we first moved out some poor quality signals, the same rate may increase a lot.

### The effect of signal sampling frequency on DF

**Fig. 9:**
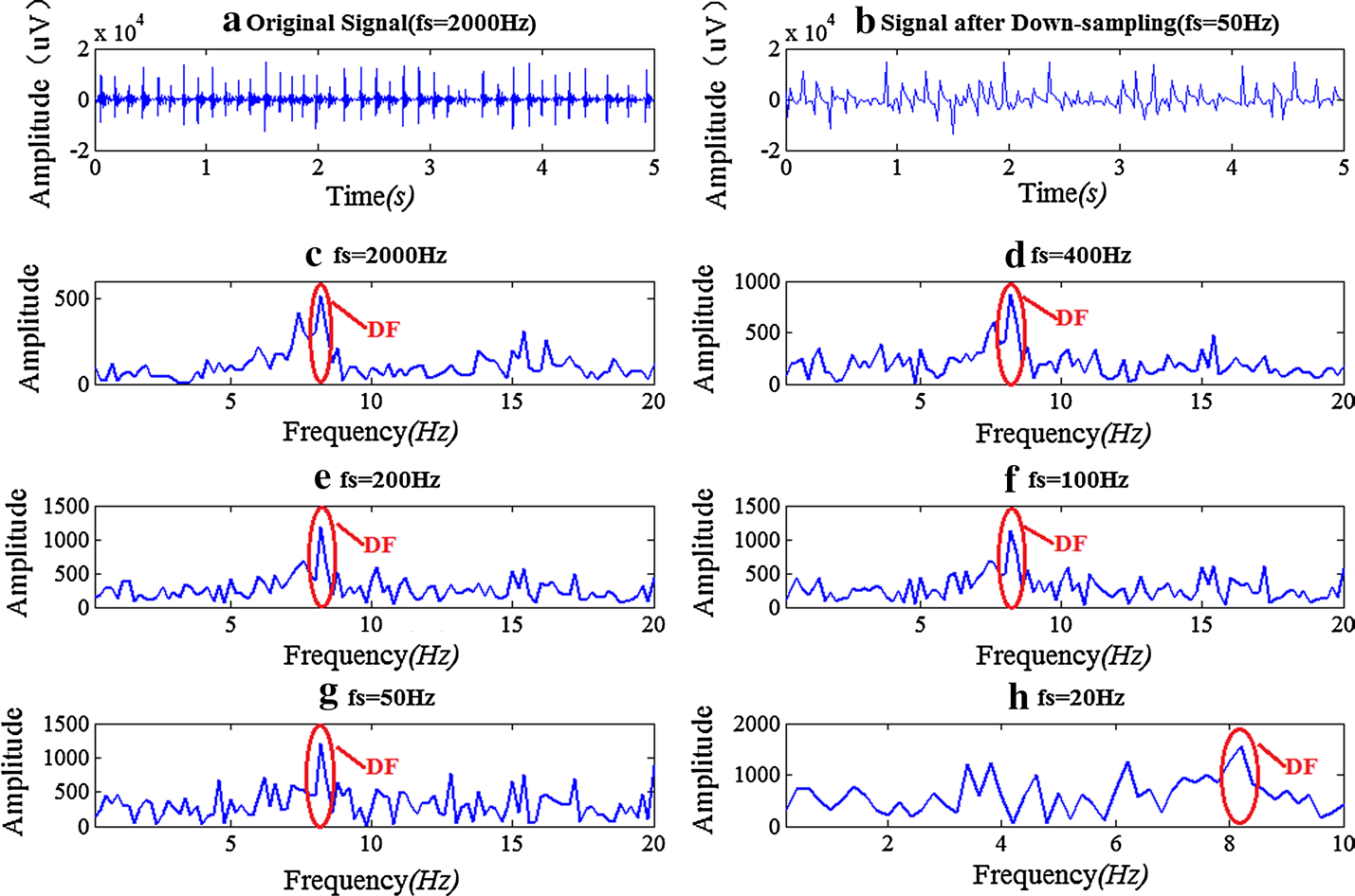
The DF result of different sampling rates for one segment of AF signal. **a** The original signal with fs = 2 kHz. **b** The signal’s morphology after down sampling to 50 Hz, which is compared with **a**. **c** The corresponding frequency spectrum and DF of **a**. When the signal of **a** down sampled to 400, 200, 100, 50, and 20 Hz, the spectrum and DFs are shown in **d**–**h**

Figure 9 shows an example about the impact on the DF results when the sampling rate of epicardial mapping signals down from 2 kHz to different rates (Fig. 9c–g). With the decreasing sampling frequency we can still get the same DF value (8.2 Hz), even though down to the extreme 20 Hz (the maximum frequency *f*_*ma*x_ < 10 Hz). Figure 9b shows the signal’s waveform in time domain when down-sampling to 50 Hz. It may be very difficult to distinguish its pattern in time domain. In comparison we can get the correct atrial activation rate from frequency domain, which is the same value achieved from the original signal in Fig. 9a.

**Fig. 10:**
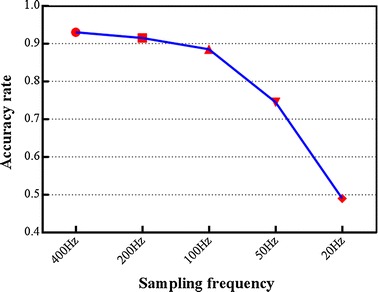
The DF accuracy rate of different sampling rate. Each segment of signal was down sampled from 2 kHz to 400, 200, 100, and 20 Hz, and the 2 kHz was the golden standard

200 segments of AF data were randomly selected from our epicardial AF signal database to do the statistical analysis. Each segment with 5 s duration was down sampled from 2 kHz to 400, 200, 100 and 20 Hz. We used the DF result of 2 kHz as the standard. The DF results of each different sampling rate were compared with this standard, and the accuracy rate was shown in Fig. 10. It is clear that with the decreasing of the sampling rate, the DF accuracy rate is reduced correspondingly. Anyhow the rate of 400 and 200 Hz both have an accuracy rate over 90 %, and 100 Hz has the accuracy rate of 87.5 %.

For one segment data of 128-channel epicardial mapping with 5 s durations, when the sampling rate is 2 kHz, the volume of data is 128 * 5 * 2000. It will be reduced to 128 * 5 * 200 (10 % of the 2 kHz situation) if the sampling rate down to 200 Hz. Down-sampling could bring a lot of benefit in saving computing time.

**Fig. 11:**
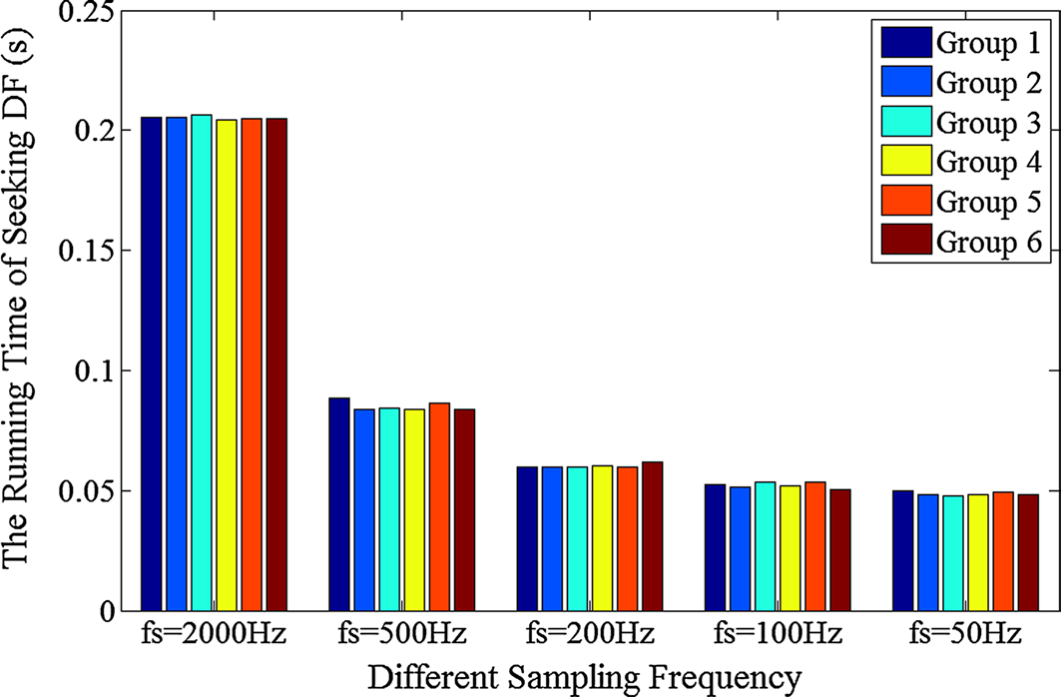
The program running times of DF seeking when using different sampling rate. Each group is 128-channel epicardial AF signal with 5 s duration, and is sampled from 2 kHz down to 500, 200, 100 and 50 Hz, respectively

Figure 11 shows the run time of seeking DF when a 128-channel epicardial AF signal down-sampling from 2 kHz to 50 Hz. We did six group experiments in total. According to the experimental results, with the decreasing of the sampling rate, the calculation time is greatly reduced. However, when the sampling rate reduces to 200 Hz or below, the shortening of calculation time becomes saturated. Combined with the accuracy rate in Fig. 10, 200 Hz may be the ideal extreme sampling rate.

### The influence of VFDs on DF

**Fig. 12:**
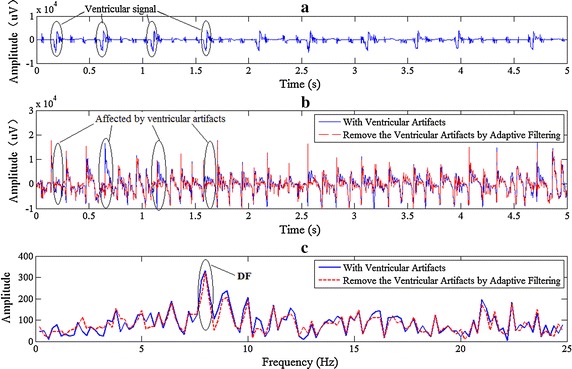
Whether removing ventricular artifact can affect the DF result in epicardial AF signal. **a** Ventricular signal from the apex of heart, 4 of 10 beats were marked out with ellipses. **b** Signal with ventricular artifacts and signal without ventricular artifacts (removed by adaptive filter). **c** Spectrum and DF

It is necessary to estimate the influence of VFDs on atrial fundamental frequency. Figure 12a shows the ventricular signal collected from the apex of one canine’s heart which can be used as a reference signal to eliminate ventricular artifacts. Graph (b) shows the comparison of original signal and the signal removed VFDs by adaptive filter. In graph (c) the frequency spectrum and DF results of these two kinds of signals are illustrated. It can be seen from graph (c) that in this case it almost has no influence on the DF result whether removing the ventricular artifact or not. The probability of getting the same results is about 87 % in our statistical analysis when using 300 segments of signal. This may be acceptable in the real-time DF mapping.

## Discussion

The preprocessing stage first used by Botteron and Smith is now considered the classical method for electrogram processing, especially for fundamental frequency detection. This paper aims to elaborate on the specific roles of those three steps for the analysis of dominant atrial rate.

In the first step the pass band range of BPF should be carefully chosen according to the signal energy distribution. We found that the best pass band for our data was 20–100 Hz, and 20–200 Hz could also get a good result, but if for other signal, this might change. Ciaccio et al. has found the optimal pass band for complex fractionated electrograms was 20–250 Hz [30]. Although it was done with bipolar signals and clinical data, its pass band was similar to our 20–200 Hz. Subsequently, the second step of taking absolute value is important in seeking DF, and we have analyzed its role from another point of view different from the paper [17]. In some cases directly doing FFT without taking absolute value and band-pass filtering can also get the correct DF result. This paper compares the two methods’ DF result on 128-channel epicardial mapping signals and finds that nearly half of the signals get the same DF result by the two methods. Nevertheless we highly recommend the nonlinear time-domain process of rectification. This is the key step to emphasize the fundamental frequency (Fig. 6). Finally, from the above results we conclude that the third step of 20 Hz LPF is completely unnecessary for the detection of dominant atrial rate.

The more regular the signal is, the more reliable the DF result will be. But for some AF signal with irregular and continuous electrical activity the DF results are not credible [9, 17]. In the experiment corresponding to Fig. 8, we find that on the whole the preprocessing method is a suitable method for AF mapping signals.

Furthermore, the sampling frequency does not affect the DF result. In real-time analysis, down-sampling could be used when doing the DF detection. It’s worth noting that the canine’s heart rate is higher than human being’s and we find that the activation rate of some signals is over 10 Hz. When the sampling frequency is decreased to 20 Hz, the largest frequency of the after-down-sampling signal is 10 Hz, it can’t get the correct DF value. This is why the accuracy rate of 50 Hz in Fig. 10 is 74.5 % but that of 20 Hz is only 49 %.

During AF ventricular artifact signals are flooded in atrial signals. If the ventricular artifacts are small, it may be unnecessary to remove ventricular artifacts when evaluating the dominant atrial rate. But with regard to body surface mapping, the impact of the ventricular signal may be taken into account because the amplitude of the ventricular signal is much larger than that of the atrial signal.

In fact, when seeking the DF value in signals with some types of artifact signals, it can also get the right result by Botteron’s approach even though the artifact is not removed. In 2013 Haissaguerre et al. used the DF of body surface mapping signal to identify localized sources of AF [31]. In their study, in order to keep the atrial component, the QRS wave is not removed even though it is much larger than the atrial wave. In this case, Botteron’s approach has the role of reducing the influence of ventricular artifact when evaluate the atrial DF. Please keep these points in mind, you will find ignoring the ventricular artifact is a feasible way for the detection of the dominant atrial rate. It is worth mentioning that the ventricular component becomes evident in unipolar electrograms with respect to bipolar recordings. However, unipolar electrograms may contain more useful information of atrial activities.

Compared to clinical recordings, canine model may have limited relevance to clinical recordings. Our model does not have changes in the substrate such as fibrosis which can lead to additional wavefronts, conduction block or discontinuities. These would add detail to the electrograms making the detection of DF more difficult.

## Conclusion

The suitability of the Botteron’s approach employed in practical applications is discussed. It can be inferred from the above that the pass band of BPF can be adjusted depending on the energy density distribution and the rectification of absolute value is important. Nevertheless the 20 Hz LPF is totally unnecessary. In addition, during AF, based on the consideration of real-time analysis in clinical practice, we can use the down-sampling method and ignore the ventricular artifacts.

## Abbreviations

DF: dominant frequency
AF: atrial fibrillation
ECG: electrogram
FFT: fast Fourier transform
BPF: band-pass filter
LPF: low-pass filter
DC: direct current
VFDs: ventricular far-field depolarizations
